# Effect of Oxidative Stress Intensity on Inflammatory, Bone Turnover, and Haemostasis Biomarkers in Patients with Spinal Osteoarthritis

**DOI:** 10.3390/life16020321

**Published:** 2026-02-12

**Authors:** Milan Mirković, Jelena Vekić, Nataša Bogavac-Stanojević, Jelena Kotur-Stevuljević, Neda Milinković, Anđelka Milić, Sanja Mirković, Ankica Vujović, Zoran Baščarević, Biljana Božić Nedeljković

**Affiliations:** 1 Institute for Orthopedic Surgery “Banjica”, 11000 Belgrade, Serbia; drckmilan@yahoo.com (M.M.); andelka.milic@iohbb.edu.rs (A.M.); zoran.bascarevic@iohbb.edu.rs (Z.B.); 2 Department for Medical Biochemistry, Faculty of Pharmacy, University of Belgrade, 11000 Belgrade, Serbia; jelena.vekic@pharmacy.bg.ac.rs (J.V.); natasa.bogavac@pharmacy.bg.ac.rs (N.B.-S.); jkotur@pharmacy.bg.ac.rs (J.K.-S.); 3 Faculty of Sport and Physical Education, University of Belgrade, 11000 Belgrade, Serbia; sanjakrcunovic@yahoo.com; 4 Clinic for Infectious and Tropical Diseases, University Clinical Center of Serbia, 11000 Belgrade, Serbia; ankica.vujovic88@gmail.com; 5 Institute of Physiology and Biochemistry “Ivan Djaja”, Faculty of Biology, University of Belgrade, 11000 Belgrade, Serbia; biljana@bio.bg.ac.rs

**Keywords:** osteoarthritis, prooxidant-antioxidant balance, vitamin D, coagulation, D-dimer

## Abstract

Osteoarthritis is associated with chronic inflammation, which contributes to a hypercoagulable state. Oxidative stress may further disrupt homeostatic balance, thereby promoting thrombotic events. This study evaluated the association between biomarkers of oxidative stress, inflammation, haemostasis, and bone metabolism in patients with spinal osteoarthritis. A total of 48 patients were included. The levels of inflammatory, bone turnover, haematological, and coagulation biomarkers were determined using standard laboratory methods. Redox status was assessed via prooxidant–antioxidant balance (PAB) and superoxide dismutase (SOD) activity. Patients with elevated PAB showed significantly higher erythrocyte sedimentation rate (ESR) (*p* = 0.005), alkaline phosphatase (ALP) (*p* = 0.003) and fibrinogen levels (*p* = 0.006) and platelet count (*p* = 0.040), along with lower 25-OH vitamin D levels (*p* = 0.045) and shortened PT (*p* = 0.008) and aPTT (*p* = 0.017). In low oxidative stress states (PAB < 100 U/L), significant correlations were observed among redox, coagulation, and bone turnover markers, whereas in high oxidative stress (PAB ≥ 100 U/L), it was characterised by predominant associations between redox and bone turnover biomarkers. Patients with grade V disc degeneration had a significantly higher probability of elevated D-dimer levels compared to those with grade IV (OR = 5.440; *p* = 0.009). In addition, elevated D-dimer levels were associated with increased ESR (*p* = 0.015), IL-6 (*p* = 0.016) and ALP levels (*p* = 0.034). The associations between biomarkers of redox status, inflammation, coagulation and bone turnover are influenced by the extent of oxidative stress. Our results suggest that PAB and D-dimer may serve as potential biomarkers for disease severity and thrombotic risk. Further studies are needed to confirm these preliminary findings.

## 1. Introduction

Osteoarthritis is a multifactorial, progressive degenerative disorder that often goes unnoticed for some time. However, significant biochemical changes have been confirmed in patients with osteoarthritis, occurring gradually until the onset of clinical manifestations [1]. Research indicates altered molecular mechanisms and specific metabolic changes, which can be confirmed by specific biomarkers [2,3,4].

Previous research has confirmed an association between vascular mechanisms and osteoarthritis and has identified impaired haemostasis, with a tendency towards hypercoagulation accompanied by an inflammatory response to the disorder [5,6,7,8,9]. The role of cartilage-derived antigens in promoting pro-coagulant activity and fibrinolysis in the pathogenesis of osteoarthritis has also been confirmed [10]. Furthermore, the close link between vitamin K-dependent proteins and skeletal development and diseases suggests that vitamin K may act as a potential mediator, bridging coagulation and bone disorders [11].

The most commonly performed coagulation tests in routine laboratories are prothrombin time (PT), activated partial thromboplastin time (aPTT), and fibrinogen. As first-line tests, together with platelet count, they guide clinicians in managing changes at the level of primary or secondary haemostasis. As in a chain reaction, diseases such as arthritis result in a loss of control mechanisms throughout the haemostatic system and, therefore, an expected change in specific tests, such as D-dimer, has also been reported [12]. This is further exacerbated by chronic inflammation, which, along with a disturbed homeostatic balance, can lead to thrombotic events. Thus, the undesirable consequences of the primary disease, in this case osteoarthritis, can also be prevented by identifying factors that may further exacerbate changes in haemostasis and inflammation parameters.

The impact and consequences of oxidative stress on the pathogenesis of osteoarthritis have been investigated and demonstrated [13,14,15]. In particular, research indicates that impaired oxidative status contributes to alterations in the intracellular redox state and oxidative modification of proteins in osteoarthritis. Conversely, antioxidant capacity is found to decrease in this condition [16]. Prooxidant–antioxidant balance (PAB) is a biomarker that universally and comprehensively reflects the contributions of both plasma oxidants and antioxidants. Thus, a higher PAB indicates increased production of reactive oxygen species, whereas lower values reflect higher antioxidant activity [17]. This is especially evident in chronic diseases [18,19,20,21,22,23,24], and the literature indicates that it can also be a significant predictor of quality of life in patients with osteoarthritis [25].

To our knowledge, the association between oxidative stress, inflammation, haemostasis, and bone metabolism impairment in osteoarthritis has not been investigated. Therefore, the aim of this study is to examine the effect of oxidative stress intensity on inflammatory, bone turnover, and haemostasis biomarkers in patients with spinal osteoarthritis.

## 2. Materials and Methods

### 2.1. Subjects

This study included 48 patients diagnosed with spinal osteoarthritis who were recruited from the Institute of Orthopaedic Surgery “Banjica”, Belgrade, Serbia. The diagnosis of osteoarthritis was based on clinical symptoms and magnetic resonance imaging (MRI). Patients were graded using the appropriate scale according to the Kellgren–Lawrence (KL) classification, which provides a quantitative score and a qualitative explanation of the degree of osteoarthritis [26]. Additionally, patients were stratified according to the Pfirrmann grading system for lumbosacral disc degeneration assessment [27]. The sample size calculation was performed using the G*Power 3.1.9.7 programme (Heinrich-Heine-Universität Düsseldorf, Düsseldorf, Germany). To determine the difference in parameter values between the examined groups with an effect size of 0.5, power (1 − β) of 0.85, significance level (α) of 0.05, and type of statistical analysis: *t*-test (Means: Wilcoxon signed-rank test, matched pairs), the calculations suggested a minimum of forty patients. To this minimum sufficient sample, 20% was added to account for unforeseen circumstances and possible dropouts.

All participants had previously undergone unsuccessful treatment with medication and physiotherapy and were prepared for surgery: spinal decompression and segmental spinal instrumentation. Patients were also using analgesics and a vitamin B complex during the course of their treatment. All participants were informed about the aim of the study, and only those who signed a consent form were included in the analysis. This study was approved by the Ethics Committee of the Institute of Orthopaedic Surgery “Banjica” under the number I-264/1, from 25 March 2021. It was conducted in accordance with the Declaration of Helsinki for human subjects [28].

### 2.2. Methods

Venous blood samples were collected in the morning after an overnight fast, prior to surgical intervention, using a standardised venepuncture procedure. All samples were processed by standard centrifugation and aliquoting procedures [29]. Biomarkers determined in serum included C-reactive protein (CRP), interleukin-6 (IL-6), procalcitonin (PCT), total calcium (Ca), alkaline phosphatase (ALP), parathyroid hormone (PTH), 25-hydroxy vitamin D (25-OH vitamin D), β-crosslaps, and osteocalcin. Redox status was assessed by determining superoxide dismutase (SOD) activity and prooxidant–antioxidant balance (PAB) level [30]. Complete blood count, including platelets, leukocytes, neutrophils, lymphocytes, and monocytes, was measured from whole blood collected in EDTA tubes. Prothrombin time (PT), activated partial thromboplastin time (aPTT), fibrinogen, D-dimer, and erythrocyte sedimentation rate (ESR) were determined from plasma collected in sodium citrate tubes. All biomarkers were measured using commercial standardised methods and reagents. Haematological parameters were analysed on the ADVIA 2120i (Siemens, Tarrytown, NY, USA) and Sysmex XN450 (Sysmex Corporation, Kobe, Japan). ESR was measured manually using the modified Westergren method. Haemostasis parameters (PT, aPTT, fibrinogen, and D-dimer) were analysed on an automated coagulometer BCS XP (Siemens, Marburg, Germany). CRP was measured by an immunoturbidimetric method. Total calcium and ALP were determined by spectrophotometric methods using a biochemical analyser Olympus AU480 (Beckman Coulter Ireland Inc., Galway, Ireland). IL-6, PCT, 25-OH vitamin D, β-crosslaps, and osteocalcin were measured using immunochemical assays on a Cobas e411 immunochemistry analyser (Roche Diagnostic GmbH, Mannheim, Germany). Redox status parameters (SOD activity and PAB) were assessed using a biochemical analyser ILab 300+ (Instrumentation Laboratory, Milan, Italy).

### 2.3. Statistical Analysis

The statistical analysis was performed using the statistical software (IBM SPSS Statistics 24). Descriptive statistics were presented as median and interquartile range (25th–75th percentile). To test differences between groups, the Mann–Whitney U test was used. Spearman’s correlation analysis was conducted to examine correlations. Binary logistic regression analysis was used to assess the predictive strength of the degree of lumbosacral disc degeneration grade in detecting increased D-dimer levels as an indicator of increased thrombotic activity. Multicollinearity among independent variables was assessed using variance inflation factors (VIFs) and tolerance values obtained from linear regression models. A VIF value > 5 and tolerance < 0.2 were considered indicative of potential multicollinearity. Data were expressed as odds ratio (OR) and 95% confidence interval (CI) (lower limit/upper limit). Statistical significance was considered for *p* less than 0.05.

## 3. Results

Demographic data of study participants are presented in Table 1. The patients had a mean age of 63 years, were generally overweight, and included more women than men (34 vs. 14). The majority of patients (45.8%) had moderate osteoarthritis severity (grade 3), while the largest proportion were classified as stage IV lumbosacral disc degeneration (54.2%) according to the Pfirrmann grading system.

The results of the laboratory parameters are shown in Table 2. The levels of the inflammatory markers were within the reference limits. Among the bone turnover biomarkers analysed, 25-OH vitamin D values were below the lower limit of the reference interval. For the haemostasis biomarkers, D-dimer values were, on average, above the clinically significant defined cut-off value.

**Figure 1:**
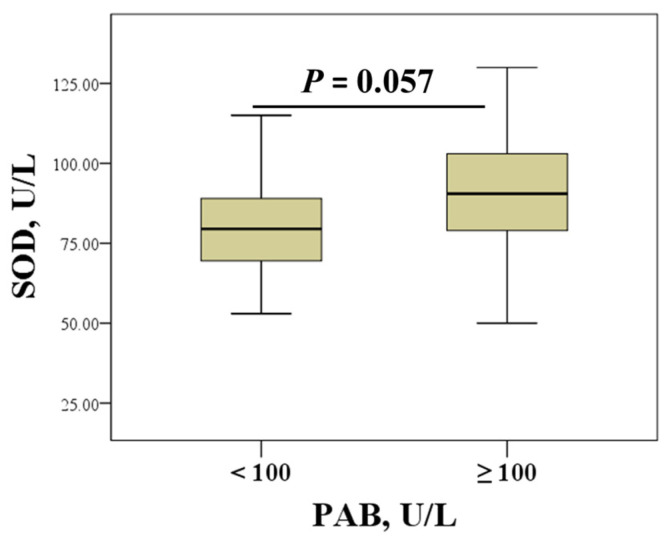
Levels of SOD according to the extent of oxidative stress defined by PAB concentrations (PAB ≥ 100 U/L indicates increased oxidative stress, and PAB < 100 U/L indicates lower oxidative stress).

Oxidative stress intensity was assessed based on the median PAB level in the entire cohort (100 U/L; interquartile range: 87–133 U/L), with values ≥ 100 U/L indicating increased oxidative stress and values < 100 U/L indicating lower oxidative stress. Table 3 presents inflammatory, bone turnover and haemostasis biomarkers stratified according to median PAB levels. The results of this analysis showed that ESR, ALP activity, platelet count, and fibrinogen levels were significantly higher in the group with elevated PAB, indicating increased oxidative stress. In addition, the level of 25-OH vitamin D was significantly lower, and PT and aPTT were shorter in the group with increased oxidative stress. As shown in Figure 1, increased PAB was associated with higher SOD activity, but this was at the border of statistical significance (*p* = 0.057).

It was also of interest to examine the mutual correlations among the investigated parameters by degree of oxidative stress (Table 4). Under conditions of lower oxidative stress, significant correlations were observed between redox and bone biomarkers. Specifically, SOD correlated negatively with β-cross laps, while PAB was positively associated with ALP activity. For inflammatory and haemostasis biomarkers, inverse associations were found between PT and both ESR and IL-6, and between aPTT and neutrophil count. Correlations between haemostasis and bone turnover biomarkers were also observed, including a positive association of fibrinogen with calcium and of D-dimer with β-crosslaps and osteocalcin. Under conditions of increased oxidative stress, PAB was negatively correlated with INR, whereas SOD was inversely associated with aPTT. Regarding bone turnover biomarkers, PAB was positively correlated with the level of 25-OH vitamin D. Furthermore, PTH levels were negatively associated with both PT and INR, while D-dimer showed a trend towards a positive correlation with ALP activity.

**Figure 2:**
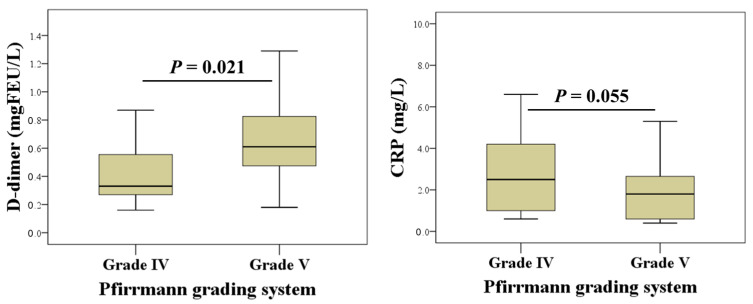
Differences in D-dimer and CRP concentrations between groups stratified by Pfirrmann grading system categories (grade IV and grade V).

Next, we performed an analysis according to the clinical characteristics of the patients and found no significant differences in the examined laboratory parameters according to osteoarthritis severity based on the Kellgren-Lawrence scale. Depending on the degree of lumbosacral degeneration, our results showed a statistically significant lower concentration of D-dimer in grade IV (median: 0.31 mgFEU/L, 25th–75th percentile: 0.23–0.63 mgFEU/L) than in grade V (median: 0.54 mgFEU/L, 25th–75th percentile: 0.24–0.67 mgFEU/L; *p* = 0.021), while the concentration of CRP was higher in grade IV (median: 2.3 mg/L, 25th–75th percentile: 1.0–3.5 mg/L) than in grade V, but this difference was at the limit of statistical significance (median: 1.2 mg/L, 25th–75th percentile: 0.5–1.9 mg/L; *p* = 0.055). The results are shown in Figure 2.

**Figure 3:**
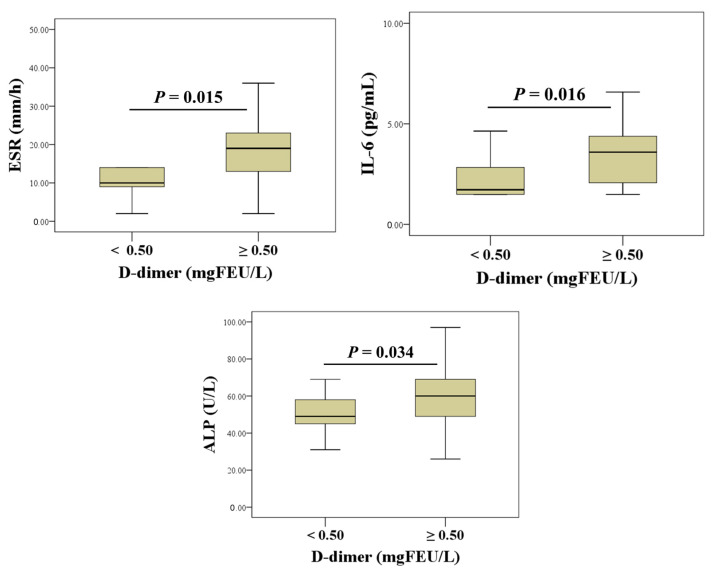
Differences between inflammatory (ESR and IL-6) and bone (ALP) biomarkers stratified by D-dimer levels (D-dimer ≥ 0.5 mg FEU/L indicates thrombotic risk).

Based on these findings, we further investigated differences in evaluated biomarkers in relation to D-dimer concentration categories (N = 21, <0.5 mgFEU/L and N = 22, ≥0.5 mgFEU/L). The results are shown in Figure 3. In the group with D-dimer concentration exceeding the clinically significant cut-off (≥0.5 mgFEU/L), we found higher ESR [14 (2–27) mm/h vs. 5.1 (3.1–7.1) mm/h; *p* = 0.015], higher IL-6 concentration [2.4 (1.5–3.3) pg/mL vs. 2.3 (1.5–3.0) pg/mL; *p* = 0.016], and higher ALP activity [62 (45–79) IU/L vs. 44 (31–57)) IU/L; *p* = 0.034].

Finally, a binary logistic regression analysis was performed to assess the association between lumbosacral disc degeneration grade and elevated D-dimer levels (≥0.5 mgFEU/L). The results showed that patients with grade V disc degeneration had 5.44-times higher odds of having an elevated D-dimer compared to those with grade IV. Importantly, the observed association remained significant after adjustment for oxidative stress biomarkers (model 3), while the significance was lost after adjustment for inflammatory (model 1) and bone turnover (model 2) markers. Of note, each model included age and BMI as covariates and was tested for multicollinearity among variables, which was not detected. In Table 5 association between lumbosacral disc degeneration grade and increased D-dimer is presented.

## 4. Discussion

In this study, we showed that patients with spinal osteoarthritis exhibit subtle changes in bone turnover and haemostasis biomarkers. Additionally, our data suggest that the relationships between biomarkers of redox status, inflammation, coagulation, and bone turnover are affected by the degree of oxidative stress.

It has been confirmed that oxidative stress significantly disrupts the body’s homeostasis and that redox changes are considered an important driver of impaired bone remodelling [31]. This effect is even more pronounced in chronic disease states associated with inflammation, such as osteoarthritis [2,3]. Additionally, activation of coagulation pathways is frequently observed in such chronic inflammatory conditions [12,14,32]. However, pronounced changes in routine biomarkers of inflammation and coagulation are usually absent in chronic diseases, most likely due to the treatment patients receive and the body’s long-term adaptation to these changes [33]. Of the biomarkers tested for bone turnover, only the values for 25-OH vitamin D were significantly below the lower limit of the reference interval. This is not unexpected, considering that the patients included in the current study were older, overweight, and in a chronic inflammatory state, all of which contribute to reduced vitamin D levels [34]. Furthermore, it has been confirmed that vitamin D supplementation does not provide any benefit in slowing osteoarthritis progression [35]. Among the coagulation parameters, only D-dimer levels were elevated above the clinically significant decision threshold for excluding thromboembolic conditions, which is consistent with the literature [10,12,36].

In our study, stratification of oxidative stress intensity according to median PAB levels revealed subtle changes in the investigated biomarkers. Notably, although within the reference range, ESR levels were significantly higher in patients experiencing increased oxidative stress. This is not unexpected, as increased oxidative stress is accompanied by activation of the body’s inflammatory responses. Furthermore, ESR itself has the potential to serve as a biomarker of stress [37]. The effect of oxidative stress is reflected in a unified and holistic response of the organism. This may also explain the higher activity of ALP, a nonspecific bone marker, in states of increased oxidative stress. In addition to the known role of this enzyme in bone turnover, anti-inflammatory mechanisms have also been described [38]. Therefore, increased levels of this enzyme can be mistakenly attributed to more active bone turnover, especially in osteoarthritis. Since total ALP activity can also be affected by hepatic function and inflammatory processes, future studies should incorporate more specific biomarkers of bone metabolism, such as bone-specific ALP and additional bone turnover markers. A similar explanation can be offered for the observed reduction in vitamin D levels in patients with elevated oxidative stress [39].

The most striking effect of oxidative stress in this study was observed on the biomarkers of coagulation. To date, few studies have evaluated the relationship between redox status biomarkers and routine haemostasis parameters in osteoarthritis [7,8,9,10]. In our study, patients with increased oxidative stress had shorter PT and aPTT, suggesting potential alterations in the coagulation pathways. However, although these differences were statistically significant, most PT and aPTT values remained within established reference ranges, which may limit the clinical relevance of these findings. Nevertheless, these results could also be influenced by potential confounding factors, including perioperative status, subclinical inflammation, recent immobilisation, or concomitant medication use. As all patients were receiving standard therapy for osteoarthritis, including analgesics and vitamin B complex, the results should be interpreted with caution, considering the potential influence of concurrent pharmacological treatment on the measured biomarkers. Taken together, our results suggest a possible association between oxidative stress intensity and coagulation changes, but this requires confirmation in future well-controlled studies. The activation of vascular mechanisms in osteoarthritis, together with the additional influence of oxidative stress, promotes a pro-coagulant and fibrinolytic state and leads to increased circulating platelet counts [5,10]. This triggers the coagulation cascade and activation of individual coagulation factors, resulting in a “laboratory phenotype” characterised by shortened coagulation times of both the extrinsic and intrinsic pathways, and increased fibrinogen concentrations. An important question remains regarding the mechanisms that maintain these haemostasis parameters within reference ranges in a chronic disease such as osteoarthritis. Evaluation of coagulation parameters could be particularly important in assessing anticoagulant therapy, which is administered to bedridden patients to prevent thrombotic events.

Our study further raises the question of whether, and to what extent, the effect of oxidative stress is undesirable. Specifically, our results revealed that significant correlations between redox biomarkers, coagulation biomarkers, and bone turnover biomarkers predominate in states of low oxidative stress, which antioxidant capacities can control. The observed negative correlation between SOD and β-cross laps, a biomarker for bone resorption, suggests that antioxidant defence mechanisms may contribute to the stabilisation of bone metabolism in osteoarthritis patients. In line with these findings, the positive correlation of D-dimer, a biomarker of fibrin degradation, with β-crosslaps, a bone resorption biomarker, and with osteocalcin, a vitamin K-dependent protein and a negative regulator of bone formation [11], suggests a simultaneous disruption in bone turnover and haemostasis balance in osteoarthritis, driven by chronic oxidative stress and inflammation. Interestingly, under conditions of high oxidative stress, when antioxidant capacities are exceeded, the correlations between biomarkers of oxidative stress and bone turnover were predominant. This finding further confirms the principal role of oxidative stress in osteoarthritis progression.

The literature on the vicious cycle between inflammation, oxidative stress, and coagulation is conflicting, particularly as it has been studied under different conditions [40]. In osteoarthritis, this relationship is further complicated by the nature of the disease, the therapy used, and the severity of the disease. Another important factor that should be considered when interpreting the observed associations is the perioperative status of patients at the time of sampling, as clinical factors and physiological stress related to surgery and anaesthesia may have influenced inflammatory, haemostasis, and oxidative stress biomarker levels. To better understand these complex interactions, we examined differences in biomarker levels stratified by clinical characteristics. However, no significant difference was found in biomarker levels based on the Kellgren and Lawrence classification system for osteoarthritis, although this classification is also used for therapy assessment [41]. Interestingly, using the Pfirrmann grading system, which is more accurate for the studied population, we found significantly higher D-dimer values and a trend towards lower CRP values in the group of patients with advanced lumbar disc degeneration. Although this discrepancy in D-dimer and CRP levels might be concerning, as both biomarkers can indicate inflammation, research suggests that this trend has predictive value for pulmonary embolism [42]. Furthermore, elevated D-dimer levels in patients with grade V degeneration could reflect underlying microvascular injury or local pro-thrombotic processes within severely degenerated discs. Indeed, we found that lumbosacral disc degeneration grade V was significantly associated with elevated D-dimer levels, even after adjusting for oxidative stress biomarkers. These findings suggest that advanced disc degeneration may be linked to a hypercoagulable state independently of systemic alterations in redox homeostasis. Elevated circulating D-dimer reflects systemic fibrin turnover and therefore cannot be attributed exclusively to local disc pathology. The observed association with Pfirrmann grade V degeneration may reflect, at least in part, disc-related processes such as chronic inflammation, microvascular alterations, and localised activation of coagulation and fibrinolysis, along with broader systemic hemostatic changes. However, given the cross-sectional design of the study, causal relationships cannot be established. In addition, D-dimer is a biomarker with low diagnostic specificity, and its levels may be influenced by age, inflammation, obesity, and vascular disease, which should also be considered when interpreting these findings. Notably, the observed association was lost after adjustment for age, BMI, inflammatory, and bone turnover markers, suggesting that these factors may modulate the relationship between D-dimer levels and advanced disc degeneration. Nevertheless, the distinct coagulation and inflammatory profile observed in patients with advanced lumbar disc degeneration warrants close monitoring and further investigation to better understand its clinical implications and potential risks.

In this study, D-dimer emerged as a meaningful biomarker, showing significant correlations and changes in relation to oxidative stress levels, as well as providing clinically relevant insights for stratifying the patient’s condition. This was evident from changes in some of the tested biomarkers observed according to the extent of risk for thrombotic conditions, as indicated by D-dimer levels. In particular, osteoarthritis patients at higher risk of thrombotic events exhibited elevated IL-6 and ESR, along with increased levels of the bone biomarker ALP. Our results are consistent with other studies showing that inflammation contributes to impaired fibrinolysis, particularly in chronic diseases [9,12,43,44]. Furthermore, elevated ALP is associated with vascular calcification [45], and this interplay between bone metabolism and coagulation may indicate a risk for cardiovascular complications.

A key strength of this study is that, for the first time, it comprehensively examined the interconnection of oxidative stress with biomarkers of bone turnover, inflammation, and coagulation in osteoarthritis. However, the cross-sectional design of the study limits causal inference. The relatively small number of subjects restricts the generalizability of the findings, leaving room for further research to draw definitive conclusions about the clinically significant effects of oxidative stress on these processes in osteoarthritis. Furthermore, the relatively small number of participants in certain subgroups, particularly those stratified by PAB levels, Pfirrmann grades, and D-dimer categories, may have limited the ability to detect statistically significant associations. Therefore, some findings should be interpreted with caution, considering the limited statistical power. In addition, the effects of oxidative stress intensity were evaluated by dichotomising PAB based on the cohort median. While this approach facilitated stratified analysis, it may have oversimplified potentially complex relationships between oxidative stress and the studied biomarkers. Therefore, the observed associations should be considered exploratory. Finally, this study assessed total ALP, a non-specific biomarker influenced by bone metabolism, hepatic function, and inflammatory processes. Future studies should also investigate specific and routine parameters of bone turnover, such as the bone isoenzyme of ALP, the N-terminal propeptide of procollagen type I, and tartrate-resistant acid phosphatase 5b, as these markers reflect significant bone response to therapy.

In conclusion, the results of this study suggest complex interactions among oxidative stress, inflammation, coagulation, and bone turnover in patients with spinal osteoarthritis, particularly those with advanced disease. The strength of associations between the evaluated biomarkers was influenced by the extent of oxidative stress. Additionally, our findings highlight PAB and D-dimer as potential biomarkers for disease progression and complications. However, the cross-sectional design of the study and the relatively small number of participants in subgroups stratified by PAB, D-dimer levels, and disc degeneration grade limit the strength of the conclusions regarding their prognostic value. Further studies with larger cohorts are required to confirm these preliminary observations and their clinical implications.

## Figures and Tables

**Table 1:** Demographic data of examined patients.

Parameter	Value *
N	48
Gender (male/female)	14/34
Age (years)	63 (56–68)
BMI (kg/m^2^)	27.2 (24.8–29.4)
Osteoarthritis severity (%)	
Mild	5 (10.4)
Moderate	22 (45.8)
Severe	19 (43.8)
Lumbosacral disc degeneration (%)	
grade IV	26 (54.2)
grade V	22 (45.8)

* Continuous variables are presented as median (25th–75th percentile), and categorical variables are presented as absolute and relative frequencies.

**Table 2:** Results of laboratory analyses in examined patients.

Parameter	Median (25th–75th Percentile)	Reference Values *
Inflammatory biomarkers		
ESR (mm/h)	14 (9–21)	1–30 male/1–20 female
Leukocytes (×10^9^/L)	7.8 (6.4–8.9)	3.4–9.7
Neutrophils (×10^9^/L)	4.8 (4.1–5.7)	2.1–6.5
Lymphocytes (×10^9^/L)	1.9 (1.4–2.4)	1.2–3.4
Monocytes (×10^9^/L)	0.4 (0.3–0.6)	0.3–0.92
CRP (mg/L)	2.1 (0.9–3.5)	0–5
IL-6 (pg/mL)	2.3 (1.5–3.9)	0–7
PCT (ng/L)	0.04 (0.04–0.05)	0.02–0.05
Bone biomarkers		
Total calcium (mmol/L)	2.5 (2.4–2.6)	2.2–2.65
ALP (U/L)	55 (46–67)	40–120
PTH (pmol/L)	3.70 (2.80–4.50)	1.59–6.04
25-OH vitamin D (nmol/L)	55 (44–72)	75–250
β-cross laps (pg/mL)	333 (292–555)	34–1008
Osteocalcin (ng/L)	23 (17–29)	11–43
Haemostasis biomarkers		
PT (s)	11.3 (10.8–11.6)	10.3–12.8
INR	0.92 (0.97–1.02)	0.8–1.2
aPTT (s)	30.1 (28.7–32.6)	25.9–36.6
Fibrinogen (g/L)	3.5 (3.2–3.9)	2.1–4.0
D-dimer (mgFEU/L)	0.51 (0.28–0.77)	<0.50
Thrombocytes (×10^9^/L)	244 (198–292)	150–450

* Reference values provided by assay manufacturers and verified in the laboratory.

**Table 3:** Effect of oxidative stress intensity on evaluated biomarkers.

Parameter	PAB < 100 U/L (N = 26)	PAB ≥ 100 U/L (N = 22)	*p*
Inflammatory biomarkers			
ESR (mm/h)	11 (8–19)	14 (12–24)	0.005
Leukocytes (×10^9^/L)	7.2 (6.0–7.9)	6.9 (6.2–8.4)	0.706
Neutrophils (×10^9^/L)	4.3 (3.4–4.9)	4.7 (3.8–5.5)	0.519
Lymphocytes (×10^9^/L)	1.7 (1.4–1.9)	1.9 (1.3–2.2)	0.742
Monocytes (×10^9^/L)	0.3 (0.2–0.5)	0.4 (0.2–0.5)	0.970
CRP (mg/L)	1.0 (0.8–2.1)	3.0 (2.1–3.3)	0.129
IL-6 (pg/mL)	3.7 (2.8–4.0)	4.5 (3.9–4.7)	0.662
PCT (ng/L)	0.040 (0.030–0.050)	0.050 (0.040–0.050)	0.723
Bone turnover biomarkers			
Total calcium (mmol/L)	2.5 (2.4–2.6)	2.5 (2.4–2.6)	0.500
ALP (U/L)	45 (34–57)	64 (54–74)	0.003
PTH (pmol/L)	3.70 (2.80–4.00)	4.50 (3.95–4.65)	0.782
25-OH vitamin D (nmol/L)	70 (55–97)	53 (48–56)	0.045
β-cross laps (pg/mL)	307 (254–337)	540 (359–644)	0.135
Osteocalcin (μg/L)	19 (13–26)	27 (21–39)	0.105
Haemostasis biomarkers			
PT (s)	11.5 (11.1–11.8)	10.8 (10.5–11.2)	0.008
INR	0.98 (0.94–1.06)	0.97 (0.94–1.01)	0.253
aPTT (s)	32.1 (30.1–36.0)	28.7 (28.3–29.9)	0.017
Fibrinogen (g/L)	3.3 (2.7–3.6)	3.6 (3.5–3.7)	0.006
D-dimer (mgFEU/L)	0.40 (0.20–0.67)	0.33 (0.29–0.50)	0.372
Thrombocytes (×10^9^/L)	221 (174–248)	271 (252–284)	0.040

Data were shown as median (25th–75th percentile) and compared by Mann–Whitney U test.

**Table 4:** Correlations of evaluated biomarkers according to oxidative stress intensity.

Condition	Biomarker 1	Biomarker 2	r	*p*
Lower oxidative stress	SOD (U/L)	β-cross laps (pg/mL)	−0.501	0.025
	PAB (U/L)	ALP (U/L)	0.476	0.022
	PT (s)	ESR (mm/h)	−0.572	0.013
	PT (s)	IL-6 (pg/mL)	−0.572	0.013
	aPTT (s)	Neutrophils (×10^9^/L)	−0.520	0.027
	Fibrinogen (g/L)	Total calcium (mmol/L)	0.510	0.013
	D-dimer (mgFEU/L)	β-cross laps (pg/mL)	0.460	0.041
	D-dimer (mgFEU/L)	Osteocalcin (μg/L)	0.494	0.023
Increased oxidative stress	SOD (U/L)	aPTT (s)	−0.584	0.028
	PAB (U/L)	INR	−0.656	0.015
	PAB (U/L)	25-OH vitamin D (nmol/L)	0.498	0.030
	D-dimer (mgFEU/L)	ALP (U/L)	0.467	0.051

**Table 5:** Association between lumbosacral disc degeneration grade and increased D-dimer.

Lumbosacral Disc Degeneration	B (SE)	Wald Coefficient	OR (95% CI)	*p*
Unadjusted	1.694 (0.649)	6.809	5.440 (1.524–19.414)	0.009
Adjusted for:				
Model 1	1.553 (0.903)	4.725	8.793 (0.804–27.754)	0.086
Model 2	2.688 (1.400)	3.687	14.705 (0.946–228.660)	0.055
Model 3	2.728 (1.093)	6.226	15.308 (1.795–130.524)	0.013

Dependent variable: D-dimer (0- < 0.5 mgFEU/L, 1- ≥ 0.5 mgFEU/L). Independent variable: 0- lumbosacral disc degeneration grade IV, 1- lumbosacral disc degeneration grade V. Model 1: CRP, IL-6, ESR, age, BMI. Model 2: ALP, β-cross laps, osteocalcin, age, BMI. Model 3: PAB, SOD, age BMI. SE, standard error; OR, odds ratio; CI, confidence interval.

## Data Availability

Data are available upon reasonable request to drckmilan@yahoo.com.
